# COVID‐19 lockdown frees wildlife to roam but increases poaching threats in Nepal

**DOI:** 10.1002/ece3.7778

**Published:** 2021-06-22

**Authors:** Narayan Prasad Koju, Ram Chandra Kandel, Hari Bhadra Acharya, Bed Kumar Dhakal, Dinesh Raj Bhuju

**Affiliations:** ^1^ Natural Resources Management Program Centre for Postgraduate Studies Nepal Engineering College Pokhara University Pokhara Nepal; ^2^ Department of Psychology University of Washington Seattle WA USA; ^3^ Department of National Park and Wildlife Conservation Ministry of Forests and Environment Babarmahal Nepal; ^4^ Ministry of Industry, Tourism, Forest and Environment Janakpur Nepal; ^5^ Resources Himalaya Foundation Kathmandu Nepal; ^6^ Mid‐Western University Surkhet Nepal

**Keywords:** biodiversity, poaching, SARS‐CoV‐2, security, wildlife crime

## Abstract

To contain transmission of COVID‐19, lockdowns or strict restrictions of people's mobility outside their residences were instituted in a majority of countries worldwide, including Nepal, where the first phase of nationwide lockdown was observed from 24 March to 21 July 2020. This sudden halt in human outdoor activities brought positive and negative impacts on forests and wildlife. We undertook a study to learn the impact of the COVID‐19 lockdown on wildlife and forests in the protected areas (PAs) of Nepal. Between July and September 2020, data on illegal activities recorded by the staff of PAs and also those reported by media were collected and analyzed. Key informant interviews (KII) were done with the park officers and security personnel by virtual communication (telephone, messenger app, and video call) to collect detailed information and for corroboration. The collected data were categorized into four groups: (a) wildlife killed, (b) wildlife injured, (c) arrest incidents related to forest crime, and (d) arrest incidents related to wildlife crime. Data from the fiscal year 2019–2020 were analyzed, comparing before lockdown and after. Among 20 PAs investigated during the lockdown, the study found substantial increases in wildlife death in two PAs, Banke National Park, and Bardia National Park. Similarly, Chitwan National Park (CNP) and Shivapuri Nagarjun National Park (SNNP) witnessed a rise in wildlife poaching. CNP and SNNP are located close to densely populated cities and also have human settlements in their peripheries. Wildlife was sighted freely roaming inside PAs during the lockdown, presumably because the absence of visitors and human activities during the lockdown decreased disturbance. Thus, the wildlife was enjoying the freedom of movement on the one hand, and on the other hand was threatened by poachers, many of whom were laid off from other activities and were taking advantage of the lapse in security.

## Graphical abstract

To contain transmission of COVID‐19, Nepal announced lockdown or strict restriction of people's mobility outside their residence from 24 March to 21 July 2020. This sudden halt in human activities brought positive and negative impacts on forests and wildlife. Wildlife crime increased during lockdown but the paradoxical situation was wildlife enjoying the freedom of movement.

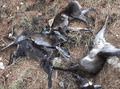

## 1 INTRODUCTION

The first infection with the SARS‐CoV‐2 virus was detected on 31 December 2019 in Wuhan, China. The World Health Organization (WHO) mission to China reported on 22 January 2020 explaining that there was evidence of human‐to‐human transmission of SARS‐CoV‐2 (WHO, 2020a). The WHO declared the outbreak a public health emergency of international concern on 30 January 2020 and officially upgraded the situation to a pandemic on 11 March 2020 (WHO, 2020a, 2020b). To contain the spread of the new virus, many countries around the world instituted a lockdown, a restrictive stay‐at‐home policy. Lockdowns contributed to an improvement in the quality of air, cleaner rivers, less noise pollution, undisturbed natural habitats, and calmer wildlife (Bulbulia et al., 2020; Rutz et al., 2020). Rutz et al. (2020) termed this effect the “Anthropause” or “the Great Pause.”

In Nepal, the first case of SARS‐CoV‐2 was recorded on 21 February 2020 followed by a second case on 23 March (Bastola et al., 2020; Sah et al., 2020; Shrestha et al., 2020). Nepal imposed a nationwide lockdown on 24 March 2020 (Ministry of Health & Population, 2020) that continued for 3 months ( March 24–21 June 2020). Restrictions were then loosened and a month‐long partial lockdown was declared from 22 June to 21 July 2020 (Ministry of Health & Population, 2020). All types of social and economic activities such as cultural festivals, social gatherings, tourism, transportation, and manufacturing were almost completely shut down. A positive result though temporary was that the lockdown cleaned the polluted skies of many capitals including Beijing, Kathmandu, and New Delhi (Mahato et al., 2020). Huang et al. (2020) reported that there was a huge decrease in NO_x_ emissions in China after the strict lockdown. The restriction also increased ozone and nighttime NO_3_ radical formation, which increased the atmospheric oxidizing capacity (Wang et al., 2021). Lockdown in Nepal coincided with the early spring season, which is the reproductive season for many wild animals including insects and large mammals (Chemineau et al., 2007, 2008). Spring is also a peak season of migratory activities, especially for birds. Chemineau et al. (2007) observed that birth peaks in mammals and birds generally occur at the end of winter–early spring, which is the most favorable period for the progeny to survive. Most species show seasonal variations in their ovulation frequency, spermatogenic activity (from moderate decrease to complete absence of sperm production), gamete quality (variations in fertilization rates and embryo survival), and also sexual behavior. “Anthropause” due to lockdown in spring may have helped wildlife reproduction by providing more undisturbed places for reproduction and mating (Rutz et al., 2020).

While there have been many research studies on the psychological and economic impacts of lockdown, only a few studies on the impact of lockdown on wildlife and biodiversity have been published. The study of biodiversity during the “quiet period” of lockdown may provide a reference for the maintenance of undisturbed ecosystems and their services. We also must think about how the COVID‐19 pandemic will impact the world's biodiversity (Corlett et al., 2020). This study aims to explore the impact of the “Great Pause” created by the COVID‐19 lockdown on wildlife in the PAs of Nepal.

## 2 METHODS

**FIGURE 1:**
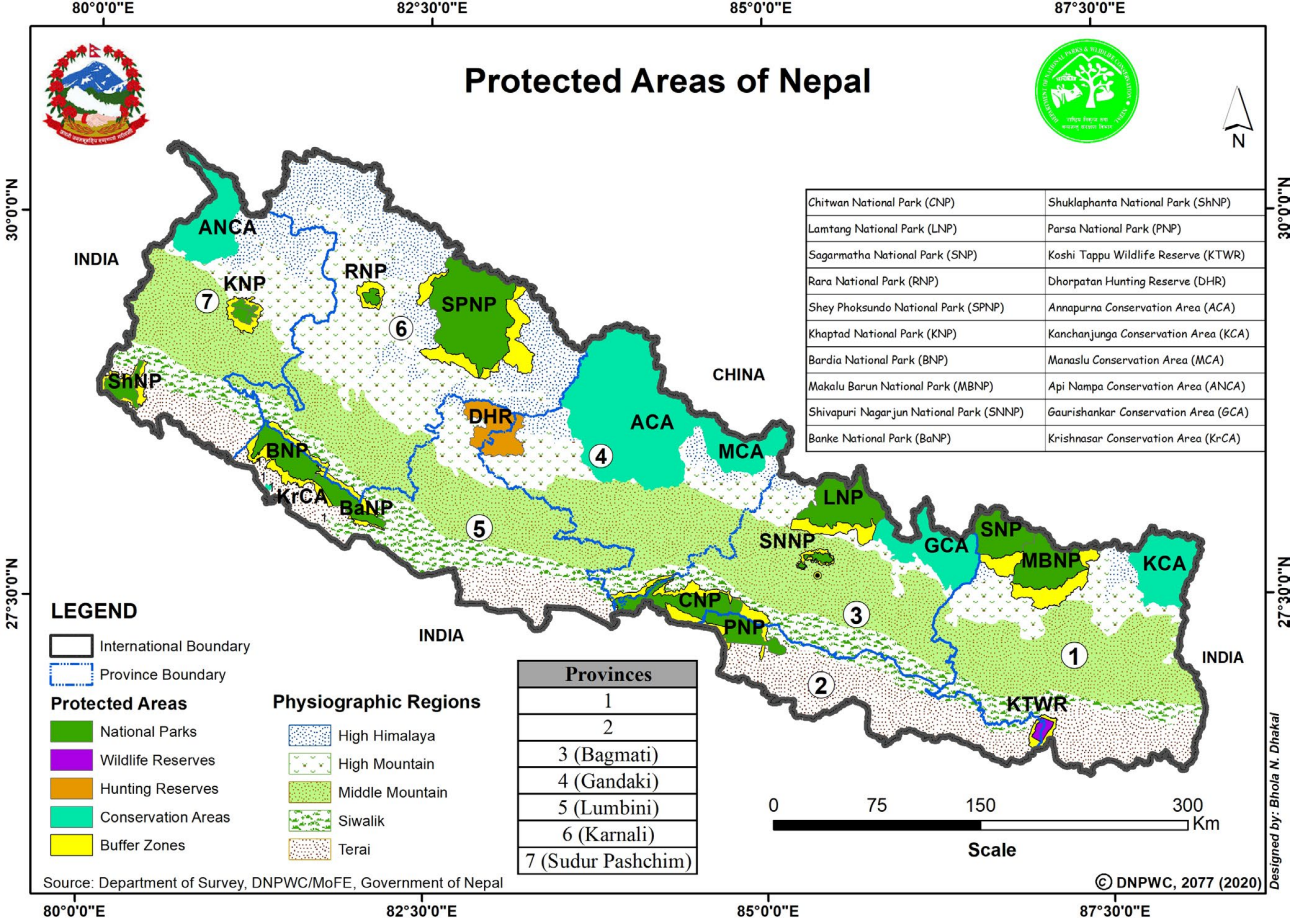
Protected areas of Nepal (*Source:* DNPWC (2020))

The study was carried out in July and September 2020. Information about various illegal activities was collected from officials of protected areas (PAs) and published reports in the media. The information included incidents of poaching, hunting, trespassing, unauthorized collection of non‐timber forest products (NTFP), fishing, and collection of fuelwood inside the PAs. Key informant interviews (KII) were done with the park officers and security personnel by telephone, messenger app, and video call to collect detailed information and also to verify data collected from various other sources. We contacted officials of all 20 PAs in Nepal via the Department of National Park and Wildlife Conservation (DNPWC) (Figure 1). The collected data were divided into four categories: (a) wildlife killed, (b) wildlife injured, (c) arrest incidents related to forest crime, and (d) arrest incidents related to wildlife crime, all within the fiscal year 2019–2020. The collected data and information were analyzed to compare two different periods: pre‐lockdown and during the lockdown. The data set was tested for normal distribution and normally distributed data was used in T tests. T tests were applied to check for statistical significance, with a threshold of 0.05 for overall significance, using the Holm–Bonferroni correction to avoid p‐value inflation.

## 3 RESULTS

### 3.1 Injured wildlife

Out of the 20 protected areas, 13 reported records of wildlife injuries not resulting in immediate death in 2019/2020 (see supplementary). The remaining seven PAs reported no records of wildlife injuries either during the lockdown or pre‐lockdown period. During the lockdown, the average number of wildlife injuries per month increased in Chitwan National Park (CNP) by 9.75%, Bardia National Park (BNP) (25.80%), Parsa National (PNP) (44.44%), and Banke National Park (BaNP) (53.84%). The average wildlife injury frequencies decreased in Suklaphanta National Park (ShNP) (40.80%), Shivapuri Nagarjun National Park (SNNP) (11.8%), and Koshi Tappu Wildlife Reserve (KTWR) (27.59%). Makalu Barun National Park (MBNP) and Rara National Park (RNP) recorded wildlife injuries only before the lockdown but no casualties during the lockdown. Sagarmatha National Park (SNP), Khaptad National Park (KNP), Langtang NP (LNP), and Api Nampa Conservation Area (ANCA) reported wildlife injuries only during the lockdown. However, there was no significant difference in the number of injured wildlife between pre‐lockdown and lockdown periods (Table 1).

**TABLE 1:** Record of wildlife injuries (not including deaths) in different protected areas during pre‐lockdown and lockdown periods in Nepal

S.N.	Location	Pre‐lockdown (mean per month, *n* = 8)	Lockdown (mean per month, *n* = 4)	*p*‐value
1	Chitwan NP	9.25	10.25	0.83
2	Bardiya NP	5.75	7.75	0.46
3	Parsa NP	1.25	2.25	0.43
4	Suklaphanta NP	8.87	5.25	0.38
5	Shivapuri Nagarjun NP	3.12	2.75	0.92
6	Banke NP	1.50	3.25	0.36
7	Koshi Tappu WR	5.87	4.25	0.41
8	Langtang NP	0	1.25	—
9	Makalu Barun NP	0.25	0	—
10	Sagarmatha NP	0	0.25	—
11	Rara NP	0.12	0	—
12	Khaptad NP	0	0.25	—
13	Api Nampa CA	0	0.25	—

### 3.2 Death of wildlife

Out of 20 PAs, 14 PAs recorded incidents of wildlife death in 2019–2020. Among them, 11 PAs had records of deaths both pre‐lockdown and during the lockdown periods. A notable large difference was observed in the number of wildlife casualties in the case of BNP and BaNP (*p* = 0.03 and 0.07, respectively). SNP had no record of deaths during pre‐lockdown, but it recorded 16 wildlife deaths (average of 4.0 per month) during the lockdown. In contrast, Makalu Barun National Park (MBNP) had two, and RNP had five incidents of wildlife death during pre‐lockdown but no death records during the lockdown period (Table 2).

**TABLE 2:** Records of deaths of wildlife in different protected areas during pre‐lockdown and lockdown periods in Nepal

S.N.	Location	Pre‐lockdown (mean per month, *n* = 8)	Lockdown (mean per month, *n* = 4)	*p*‐value
1	Chitwan NP	3.25	1.75	0.29
2	Bardiya NP	7.37	17.25	0.07
3	Parsa NP	1.5	1	0.65
4	Langtang NP	2.25	3	0.70
5	Suklaphanta NP	5.37	1.5	0.18
6	Shivapuri Nagarjun NP	0.25	1.5	0.16
7	Banke NP	2.875	8	0.03
8	Khaptad NP	0.125	0.25	0.62
9	Api Nampa CA	0.25	1	0.34
10	Krishnasar CA	0.625	1	0.72
11	Kanchenjunga CA	0.25	0.25	1
12	Sagarmatha NP	0	4	‐
13	Makalu Barun NP	0.25	0	‐
14	Rara NP	0.65	0	

**FIGURE 2:**
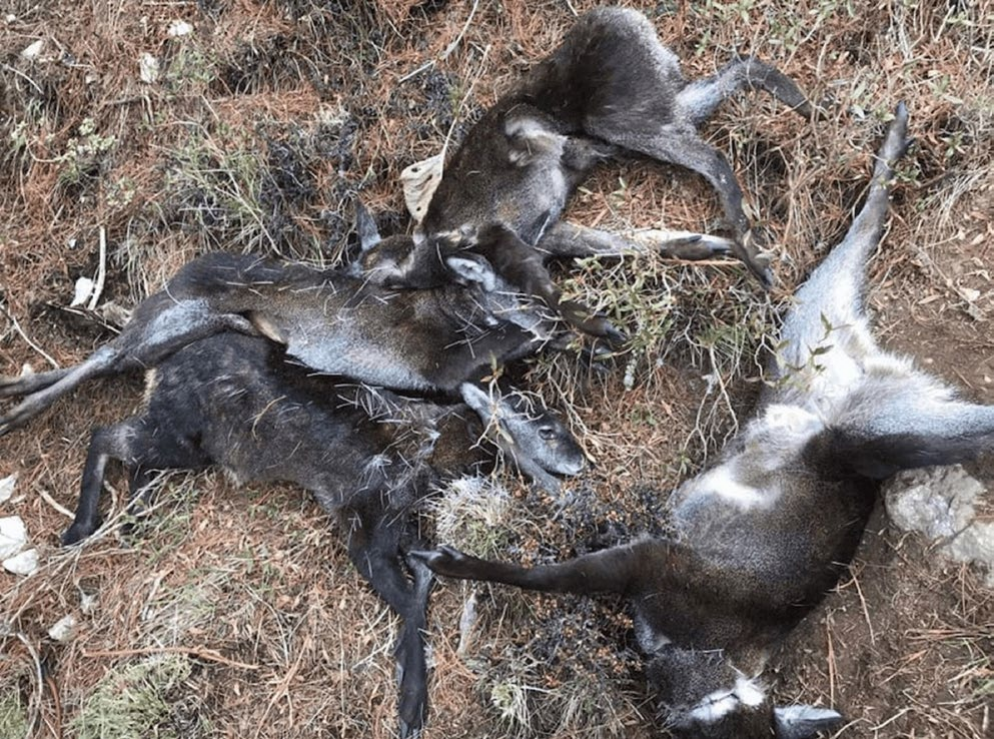
Musk deer killed in snares in Sagarmatha NP during COVID‐19 lockdown (Photograph credit: *Dawa Nuru Sherpa*)

Six musk deer were killed by poachers using snares in SNP on 26 April 2020, the 32nd day of the lockdown (Figure 2). It was inferred that the poachers took advantage of the COVID‐19 lockdown which forced people to confine themselves in their homes, including national park staff and forest guards (Nepali Times, 2020).

A similar situation may have applied in other PAs. In PNP during the lockdown, there was a deadly exchange of fire between the army and poachers in which one poacher was killed. In BNP on 27 March 2020 poachers killed an elephant, and there was an exchange of fire between poachers and the Nepal Army, which led to the death of a poacher and the injury of army staff. These incidents suggest that park management and security personnel faced huge poaching pressure both inside and outside the protected area during the lockdown.

### 3.3 Forest‐related crime arrest cases

Forest‐related crime comprises illegal trespass inside the protected area, illegal logging, and illegal collection of medicinal herbs and sources of food (wild mushroom, bamboo shoot, ferns, etc.), and other non‐timber forest products (NTFP) from PAs. In the fiscal year 2019–2020, 12 PAs did not record any crime related to forests. Among the remaining eight PAs, CNP had a significant increase in forest‐related crime (*p* = 0.02). On average in CNP, 26.5 crime cases per month were reported pre‐lockdown, which jumped to 182.25 cases per month during the lockdown. Similarly, incidents increased by 5.68 times in SNNP (*p* = 0.02, Table 3).

**TABLE 3:** Forest‐related crime in different protected areas during normal and lockdown periods in Nepal

S.N.	Location	Pre‐lockdown (mean per month, *n* = 8)	Lockdown (mean per month, *n* = 4)	*p*‐value
1	Chitwan NP	26.50	182.25	0.02
2	Bardia NP	7.5	3.67	0.72
3	Parsa NP	4.12	0.33	0.32
4	Makalu Barun NP	3.25	1.67	0.61
5	Shey‐Phoksundo NP	1.6	6.5	0.11
6	Sagarmatha NP	1.375	0.5	0.67
7	Shivapuri Nagarjun NP	29.375	166.75	0.02
8	Banke NP	2.375	3.5	0.53

The Assistant Warden of CNP Mr. Rishi Ranabhat reported that the park faced extreme pressure of illegal activities during the first month of lockdown. The wild animals were freely roaming inside the park, presumably because there were no visitors, and other human activities also sharply decreased. He said the patrolling team of the park had observed 32 rhinos while traveling 17 kilometers from Sauraha to Kasara, and the park staff counted 600 spotted deer in a single spot at Bhimlephant. Similarly, 42 blue bulls were spotted at one spot in grassland. Mr. Ranabhat reported that the record of illegal cases in CNP in the first month of the lockdown was higher than the total cases recorded in the previous 11 months. A total of 483 cases of illegal acts were filed before lockdown between April 2019 and March 2020, whereas 514 cases were filed in the first month of the lockdown period. CNP also recorded cases of poachers killing an elephant and three critically endangered gharials within the first 10 days of the lockdown.

**FIGURE 3:**
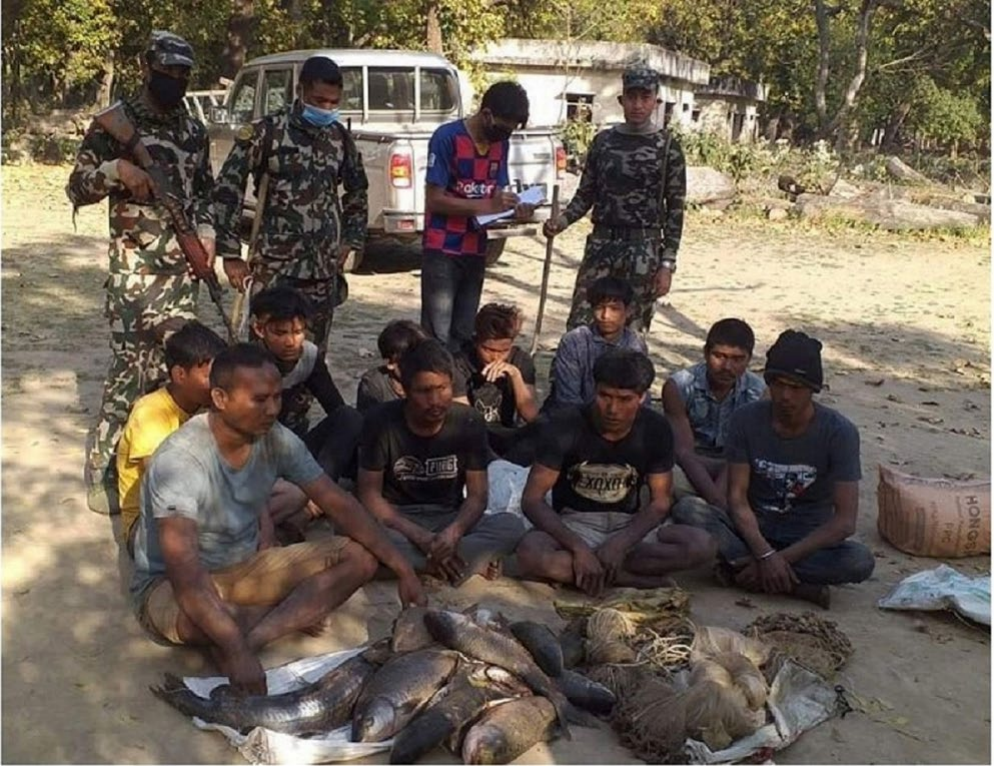
A group of people arrested by the patrolling team with a gill net and illegally caught fish during lockdown (Photograph credit: Setikali Express, Kanchanpur, *Nepal*)

At CNP, the trespass increased threefold during the lockdown as compared with pre‐lockdown. Illegal entry into the park also increased during strict lockdown after local people returned to their villages from cities. People were active in the park, especially in illegal fishing. The record of illegal fishing was four times higher than normal during the lockdown. Generally, CNP arrests 10–15 fisherman with gill nets per month but during the lockdown (Figure 3), more than 150 fishermen with gill nets were arrested and cases filed. Gill nets can capture more than 200kg of fish in one sitting and they may also capture crocodilians, especially the critically endangered Gharial (CNP, 2019; Neupane et al., 2020). In response, a collaborative effort with Nepal Army and Buffer Zone Committee members was done, and the patrolling rates were increased threefold over pre‐lockdown to control illegal fishing and poaching (as per communication with Mr. Rishi Ranabhat).

Mr. Keshav Dodhari, an officer of SNNP, experienced similar incidents at Shivapuri Nagarjun NP. According to Mr. Dodhari, 50–60 people were arrested daily at SNNP in the initial days of the lockdown. Most people entered the park illegally to collect fuelwood and mushrooms. Forests near Tokha, Sundarijal, and Budhanilkantha are well‐known areas for mushroom collection, which was also practiced before the declaration of the PA. He said that cases of logging also increased in SNNP by four times compared to pre‐lockdown. Some groups of people were also arrested while setting nets and snares. SNNP also increased patrolling 2–3 times to control the illegal activities inside the park after these arrests.

Reports of logging and illegal smuggling of timber during the lockdown were also reported outside the protected area. The Nepal Army confiscated illegally collected timber in 10 districts, viz. Siraha, Kapilvastu, Rupandehi, Palpa, Kanchanpur, Kailali, Bara, Morang, Arghakhanchi, and Ilam. Mr. Dakshya Kumar Basnet, District Superintendent of Police (DSP) of Nepal Armed Police Force (APF) of Kailali district reported that incidents of logging and timber smuggling greatly increased in the district during the lockdown. Officers of the Division Forest Office in Kalali confirmed that they had arrested 10 different teams of smugglers from Bhajani, Godawari, Bardagoriya, Lamki, and Ghodaghodi area during the first month of the lockdown (Aryal, 2020; Ghimire, 2020; Himalayan News Service, 2020b; RSS, 2020). The spokesperson of Dhading District Police Office, Mr. Rupak Khadka, and Assistant DFO Mr. Loknath Lamsal provided the information that the Dhading DFO arrested a group of people who were smuggling more than 73 cubic feet of Sal (*Shorea robusta*) timber during the initial days of lockdown. Similarly, AFO and an Inspector of District Armed Police Force (APF) in Sunsari Mr. Krishana Dhakal jointly arrested two teams of smugglers with more than 120 cubic feet of Sal timber in a truck and tractor that were leaving Sunsari district. Chairperson of Jana Jagriti Community Forest, Kanchanpur district reported that they arrested two teams of smugglers who were cutting logs in their community forest. Smugglers had felled five trees and hidden six logs. Such incidents in Kanchanpur increased in the initial 2 months of the lockdown.

### 3.4 Wildlife crime‐related arrests

Only six PAs had a record of arrests of people involved in wildlife crime in the fiscal year 2019–2020. During the lockdown, records of wildlife crime significantly increased in CNP (*p* = 0.02). The record was very high in SNNP too, but there was no record of any such cases during pre‐lockdown. Wildlife‐related crime rates were also higher in BNP and SNP, but these increases were not statistically significant (Table 4). Wildlife crimes were also reported from outside the PAs during the lockdown. Divisional Forest Officer of Bajura, Mr. Bhim Prasad Kandel reported that illegal activities inside the community and national forests greatly increased in the district during the lockdown. DSP Bajura, Mr. Tanka Prasad Acharya added that many local poachers entered the forests to capture and kill Impeyan pheasant (*Lophophorus impejanus*), northern red barking deer (*Muntiacus* *vaginalis*), and other wildlife. The bushmeat was consumed for personal use and also sold in the market. Similar reports were received from Morang, Sunsari, Mahotari, Bardiya, and Surkhet districts. Mr. Surendra Subba, Chairperson of Arjundhara Community Forest, Jhapa district (border with India) reported that a group of poachers and smugglers were active during lockdown for logging and poaching. Mr. Khem Sitaula, Chairperson of Jamunakhai Community Forest, Jhapa, added that they could not control smuggling and poaching effectively during the lockdown, even though they increased the frequency of patrols. Jhapa district suffered from cross‐border effects in poaching and illegal activities during the lockdown.

**TABLE 4:** Wildlife crime in different protected areas during normal and lockdown periods in Nepal

S.N.	Location	Pre‐lockdown (mean per month, *n* = 8)	Lockdown (mean per month, *n* = 4)	*p*‐value
1	Chitwan NP (CNP)	14.75	90.50	0.02
2	Bardia NP (BNP)	1.875	5.5	0.23
3	Parsa NP (PNP)	1.25	0.33	0.54
4	Banke NP (BaNP)	2.125	1.333	0.48
5	Sagarmatha NP (SNP)	0.375	2.333	0.20
6	Shivapuri Nagarjun NP (SNNP)	0	7	—

## 4 DISCUSSION

Overall, it appears that all forests in Nepal including community forests were under tremendous pressure from poaching and smuggling during the lockdown (Himalayan News Service, 2020a; Sharma, 2020). The reduction in law enforcement and patrolling caused a surge of illegal killing of wildlife and activities disturbing wildlife (Aditya et al., 2021; Jones & McGinlay, 2020; Manenti et al., 2020). Aditya et al. (2021) also reported an increase in wildlife crime in India, reporting 117 pangolin seizures between January 2018 and August 2020 from 319 states across India. The pandemic emphasized the interconnectedness of people and ecosystems, and the multidimensional interdependencies between the sustainability principles (Smith et al., 2021). In our study, two PAs, CNP, and SNNP witnessed a large increase in incidents. Later, patrolling was increased, yet there were still incidents of poaching and smuggling. As both the PAs are located close to heavily populated cities and have some human habitation inside the PAs, the illegal activities could have occurred from settlements used as hiding places during the lockdown. Regarding the six musk deer killed in SNP, Pokharel (2020) opined that some people might have become involved in poaching after they lost their income due to the lockdown. Moreover, security personnel (army and police) were diverted to other towns to regulate lockdown. A lackluster intelligence network because of stressful times at the beginning of the pandemic may also have contributed to poaching. This is a great lesson learned to intensify the poaching and illegal activities inside the forest and protected area during the pandemic (RSS, 2020; Saeed et al., 2020).

In contrast, the combination of the lockdown and daily announcements of COVID‐19 cases and casualties in media (including social media) also brought positive impacts in the form of the Great Pause of outdoor movement of people. This helped to reduce not only air pollution and noise pollution, but perhaps also created benefits for wildlife. Manenti et al. (2020) reported positive effects of lockdown on wildlife conservation in Italy with an increase in species richness in temporarily less‐disturbed habitats, higher breeding success of an aerial insectivorous bird, and reduction in roadkill of both amphibians and reptiles. Social media around the world shared photographs of surprising wildlife encounters during the lockdown, especially in urban environments (Szozda & Shutterstock, 2020). People reported sightings of such rare animals as pumas in downtown Santiago, Chile (FRANCE^24^, ^2020^), dolphins in atypically calm waters in the harbor of Trieste, Italy (Rutz et al., 2020), jackals seen in broad daylight in urban parks in Tel Aviv, Israel (Dickerman, 2020), and herds of wild goats (*Capra* spp.) in towns in North Wales (BBC, 2020a) and Turkey (Reuters/VoA, 2020). Spotted deer (*Axis axis*) were seen in Colombo, Sri Lanka (Malaka, 2020), lions (*Panthera leo*) in Africa were noticed occupying highways (BBC, 2020b), barking deer (*Muntiacus reevesi*) were seen on the highways of Japan (Evans, 2020), deer and civets (*Viverra* sps) roamed in urban areas of Europe and India (Krishnankutty, 2020). In Nepal, there were not only more animals observed than usual in Kathmandu valley, but also some unexpected visitors, such as white Egyptian vulture (*Neophron percnopterus*), seen for the first time in 20 years during lockdown (Newslaya, 2020), and a report of common leopard (*Panthera pardus*) seen on the Tribhuvan University premises in Kirtipur, Kathmandu (Kirtionline, 2020).

It would be too early to conclude that the observations of wild animals seen in cities of Europe, Africa, and Asia, including Kathmandu, were driven lockdown and reduced human movement, as has been hyped by social media. However, it can be hypothesized that the impact of human activities on wildlife has become more pervasive than ever before, and it will be important to study its complex effects on wildlife behavior and movement.

## 5 CONCLUSION

We conclude that protected areas near human settlements in Nepal have experienced a negative impact from lockdown since forest and wildlife‐related crimes were significantly increased. Death of wildlife inside PAs also significantly increased in BNP and BaNP, though the injury records of wildlife did not change much between pre‐lockdown and lockdown. During the strict lockdown, wildlife was sighted more frequently and they were roaming freely. However, the diversion of security personnel gave opportunities to poachers and timber thieves, some of whom may have been urbanites laid off from their jobs.

A special arrangement of surveillance involving local communities could help to mitigate such illegal activities during any future lockdowns.

## CONFLICT OF INTEREST

None declared.

## AUTHOR CONTRIBUTIONS

**Narayan Prasad Koju:** Conceptualization (lead); Data curation (equal); Formal analysis (lead); Funding acquisition (lead); Investigation (equal); Methodology (lead); Resources (lead); Supervision (equal); Validation (lead); Visualization (equal); Writing‐original draft (lead); Writing‐review & editing (lead). **Ram Chandra Kandel:** Conceptualization (supporting); Data curation (lead); Formal analysis (supporting); Investigation (supporting); Methodology (equal); Project administration (lead); Resources (supporting); Supervision (equal); Validation (equal); Writing‐original draft (supporting); Writing‐review & editing (supporting). **Hari Bhadra Acharya:** Conceptualization (supporting); Data curation (lead); Formal analysis (supporting); Investigation (supporting); Project administration (supporting); Validation (equal); Writing‐original draft (supporting); Writing‐review & editing (supporting). **Bed Kumar Dhakal:** Data curation (equal); Investigation (supporting); Methodology (supporting); Validation (equal); Writing‐original draft (supporting); Writing‐review & editing (supporting). **Dinesh Raj Bhuju:** Conceptualization (lead); Data curation (supporting); Formal analysis (equal); Funding acquisition (lead); Investigation (equal); Methodology (equal); Project administration (equal); Supervision (lead); Validation (lead); Writing‐original draft (equal); Writing‐review & editing (equal).

### OPEN RESEARCH BADGES

This article has been awarded Open Data, Open Materials, Preregistered Research Designs Badges. All materials and data are publicly accessible via the Open Science Framework at [https://osf.io/awmj4/files/].

## Data Availability

The data associated with this manuscript are available at: https://datadryad.org/stash/share/EzQdl80wDM2yBS0‐bwisnsRTTSLX3XDg7Rl2gUzQxIA.
